# Effects of a Behavioral Weight Loss Intervention and Metformin Treatment on Serum Urate: Results from a Randomized Clinical Trial

**DOI:** 10.3390/nu13082673

**Published:** 2021-07-31

**Authors:** Jiun-Ruey Hu, Hsin-Chieh Yeh, Noel T. Mueller, Lawrence J. Appel, Edgar R. Miller III, Nisa M. Maruthur, Gerald J. Jerome, Alex R. Chang, Allan C. Gelber, Stephen P. Juraschek

**Affiliations:** 1 Department of Medicine, Vanderbilt University Medical Center, Nashville, TN 37232, USA; jiun-ruey.hu@yale.edu; 2 Welch Center for Epidemiology, Prevention, and Clinical Research, Johns Hopkins Medical Institutions, Baltimore, MD 21287, USA; hyeh1@jhmi.edu (H.-C.Y.); noeltmueller@jhu.edu (N.T.M.); lappel@jhmi.edu (L.J.A.); ermiller@jhmi.edu (E.R.M.III); maruthur@jhmi.edu (N.M.M.); agelber@jhmi.edu (A.C.G.); 3 Department of Kinesiology, Towson University, Towson, MD 21252, USA; gjerome@towson.edu; 4 Division of Nephrology, Geisinger Health, Danville, PA 17822, USA; achang@geisinger.edu; 5 Division of Rheumatology, Johns Hopkins University School of Medicine, Baltimore, MD 21224, USA; 6 Division of General Medicine and Primary Care, Beth Israel Deaconess Medical Center, 330 Brookline Avenue, CO−1309, #216, Boston, MA 02215, USA

**Keywords:** uric acid, serum urate, weight loss, metformin, randomized clinical trial

## Abstract

**Background:** Lower body mass index (BMI) has been associated with lower serum urate (SU), but only in observational studies. We sought to determine the effects of behavioral weight loss and metformin treatment on SU in a randomized trial. **Methods and Findings:** The Survivorship Promotion In Reducing IGF-1 Trial (SPIRIT) was a parallel three-arm randomized controlled trial of overweight/obese adult cancer survivors without gout at a single center in Maryland, United States. Participants were randomized to: (1) coach-directed weight loss (behavioral telephonic coaching), (2) metformin (up to 2000 mg daily), or (3) self-directed weight loss (informational brochures; reference group). SU and BMI were assessed at baseline and at 3, 6, and 12 months post-randomization. The 121 participants had a mean ± standard deviation (SD) age of 60 ± 9 years, 79% were female, and 45% were Black. At baseline, BMI was 35 ± 5 kg/m^2^, and SU was 5.6 ± 1.3 mg/dL. Compared to the self-directed group, at 12 months, the coach-directed group reduced BMI by 0.9 kg/m^2^ (95% confidence interval (CI): −1.5, −0.4) and metformin reduced BMI by 0.6 kg/m^2^ (95% CI: −1.1, −0.1). However, compared to the self-directed group, the coach-directed group unexpectedly increased SU by 0.3 mg/dL (95% CI: 0.05, 0.6), and metformin non-significantly increased SU by 0.2 mg/dL (95% CI: −0.04, 0.5); these effects were attenuated when analyses included change in estimated glomerular filtration rate (eGFR). **Conclusions:** In this randomized trial of cancer survivors without gout, reductions in BMI either increased or did not change SU, potentially due to effects on eGFR. These results do not support a focus on BMI reduction for SU reduction; however, long-term studies are needed. **ClinicalTrials.gov Registration**: NCT02431676.

## 1. Introduction

Gout is a painful and debilitating inflammatory arthropathy resulting from monosodium urate crystal deposition, affecting over 3.9% of adults in the United States [1,2]. Obesity is strongly related to hyperuricemia and gout [3] in both men and women [4,5]. A lower body mass index (BMI) has been associated with a lower risk of gout [6] and lower urate levels [7,8,9,10,11,12,13,14]. These observational data formed the basis of the conditional recommendation by the 2020 American College of Rheumatology guidelines for reducing weight to manage gout among overweight/obese patients [15]. However, evidence for this recommendation was derived from surgical gastroplasty interventions [7,11] and from observational cohort studies [12]. There is limited, if any, trial evidence examining the impact of BMI reduction upon SU [14,16].

SPIRIT (the Survivorship Promotion In Reducing IGF-1 Trial) was a randomized controlled trial of cancer survivors that compared the effects of a coach-directed weight loss intervention versus daily metformin versus a self-directed reference group on levels of insulin growth factor-1 (IGF-1) over a 1-year period [17,18]. These interventions were selected based on hypotheses that weight loss [19] and metformin might reduce cancer risk [20] via IGF-1, a biomarker associated with cancer development. Moreover, some observational studies suggest that metformin might reduce SU [21].

The present study is a secondary analysis of SPIRIT to assess the effect of the coach-directed intervention or metformin on SU compared to the reference group. We hypothesized that an intentional reduction in BMI would reduce SU levels within 3-months of starting the intervention and that these reductions would be maintained over time. To further understand the relationship between BMI and eGFR, we secondarily performed a mediation analysis, examining whether these effects were driven by changes in kidney function.

## 2. Patients and Methods

### 2.1. Study Design & Intervention

SPIRIT was a three-arm parallel, randomized controlled trial conducted in 2015–2017 in which cancer survivors were randomized to one of three arms: a coach-directed weight loss intervention, metformin, or self-directed weight loss (reference group) [17,18]. Randomization was computer-generated and stratified by BMI (≥30, <30 kg/m^2^), race (Black, non-Black), and were equally allocated to the 3 study arms with randomly-selected block sizes of 3 and 6. Participants in the self-directed arm received written information about weight management at study initiation. Participants in the coach-directed arm received phone calls from a behavioral coach that focused on reducing calorie intake and increasing physical activity, with calls initially placed weekly and then monthly, and web-based reporting of home weights. Participants in the metformin arm were provided up to 2000 mg/day based on tolerance, kept a pill diary, and received calls, initially placed weekly during the titration period and then as needed. The primary outcome of the parent study was insulin growth factor-1 (IGF-1), a biomarker associated with cancer development. Although it was not possible to mask the trial participants or intervention staff to the randomization assignment, staff involved in follow-up data collection and lab measures were masked to the randomization assignments. Although patients were not directly involved in the design and conduct of the study and the choice of outcome measures, our research center offers multiple events per year to allow patients to provide feedback on research activities. All participants provided written informed consent. The study was approved by the Johns Hopkins Institutional Review Board (IRB #00035653), and funded by the Maryland Cigarette Restitution Fund. Datasets are available upon reasonable request. The trial is registered at ClinicalTrials.gov under NCT02431676.

### 2.2. Study Population

Between June 2015 and December 2016, we recruited participants in Baltimore who were ≥18 years old, previously diagnosed with a malignant solid tumor, and who had completed their oncologic treatment (surgery/chemotherapy/radiation) at least three months prior to enrollment [22]. Exclusion criteria included medication-treated diabetes mellitus or hemoglobin A1c ≥ 7%, use of metformin within the prior 3 months, use of weight loss medications within the prior 6 months, prior or planned bariatric surgery, current or planned enrollment in a weight loss program, chronic kidney disease (eGFR < 45 mL/min/1.73 m^2^), hepatic disease (alanine aminotransferase or aspartate aminotransferase ≥ 2 times upper limit of normal, or self-reported liver disease), or heavy alcohol consumption (>14 drinks/week) (Table S1).

### 2.3. Study Outcome

The study outcome of the present report was fasting SU collected at baseline and at 3, 6, and 12 months after randomization. Blood was collected at each visit in a serum separator tube, allowed to clot for 20 min at room temperature, then centrifuged for 15 min and sent to Quest Diagnostics. SU and serum creatinine were quantified by spectrophotometry. Estimated glomerular filtration rate (eGFR) was calculated using the Chronic Kidney Disease Epidemiology Collaboration (CKI-EPI) equation [23]. Enrollment, baseline, and follow-up visits took place at ProHealth, a community-based ambulatory research facility in Baltimore (Woodlawn), MD, USA.

### 2.4. Other Covariates

Variables collected at baseline included age, sex, race, blood pressure, BMI, total cholesterol and high density lipoprotein cholesterol, eGFR, alcohol use, and primary cancer type. Each visit involved questionnaires, phlebotomy, stool and urine collection, and measurement of height, weight, and blood pressure. History of gout was not collected. High SU was defined as >7 mg/dL in men and >6 mg/dL in women.

### 2.5. Statistical Analysis

In the main study design, a total sample of 120 individuals was calculated to detect a 10% reduction in the primary outcome (IGF-1) with 80% power using a 2-sided z-test with alpha of 0.025. Baseline characteristics were summarized overall and by intervention assignment. Continuous variables were summarized as means and standard deviations (SD); categorical variables were summarized as proportions. Changes in SU, BMI, and eGFR were compared to baseline at 3-, 6-, and 12-month, using generalized estimating equations (GEE) with a Huber and White robust variance estimator which assumed an exchangeable working correlation matrix, which included a SU and visit-month interaction term.

Comparisons between arms were also performed using GEE (described above). Specific time points in the study (3, 6, and 12-months) were determined with a visit coefficient adjusted for baseline SU. We also performed a combined analysis, which pooled data across all 3 follow-up visits following a repeated measures analysis that treated each of the 3 follow-up visits equally.

To explore the relationship of change in BMI with change in SU, we conducted mediation analyses, using the Baron and Kenny approach [24]. The cross-sectional relationship between SU and BMI across all visits was examined by linear regression with BMI divided into categories of BMI change from baseline: −2, −1, −0.5, +0.5, and +1 kg/m^2^ respectively. Similarly, the cross-sectional relationship between SU and eGFR were examined in categories of change from baseline, namely, −10, −5, 0, +5, and +10 mL/min/1.73 m^2^. This was plotted with the addition of a Lowess smoother. All analyses were performed in Stata 15.1 (Stata Corporation, College Station, TX, USA). Statistical significance was defined as *p* ≤ 0.05 without Bonferroni correction.

## 3. Results

### 3.1. Baseline Characteristics

There were 121 participants in the trial, with a mean (SD) age of 60 years (9.0) and mean (SD) BMI of 34.9 kg/m^2^ (5.4). Of trial participants, 79% were female and 45% were African-American. Participant characteristics were balanced across intervention assignments (Table 1).

### 3.2. Change in Weight from Baseline

**Figure 1:**
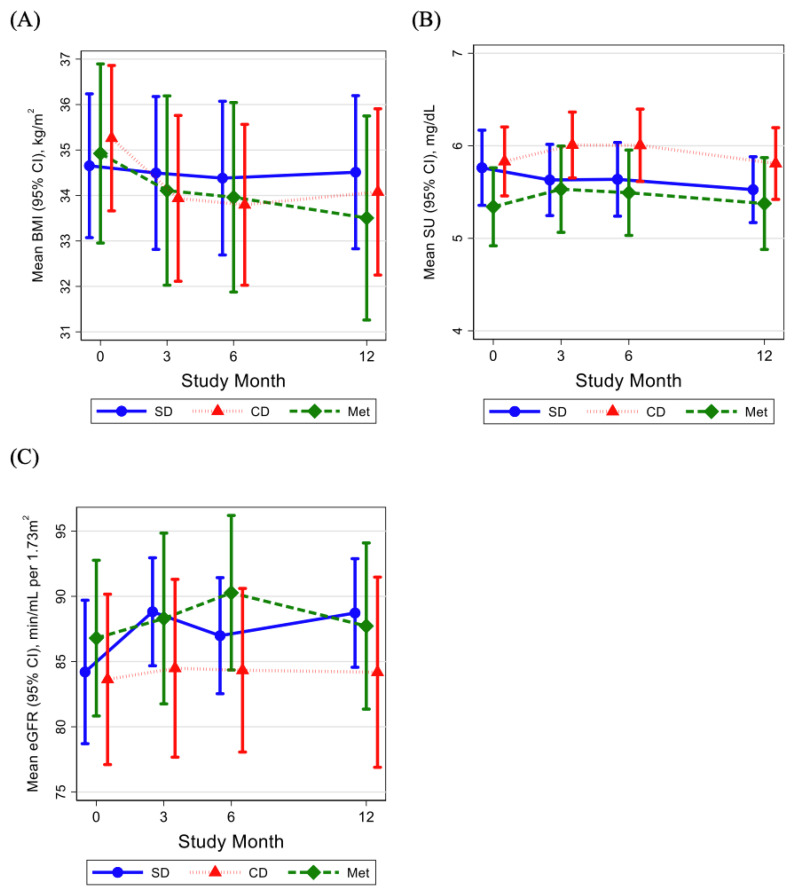
(**A**) Mean body mass index (BMI, in kg/m^2^) and (**B**) mean serum urate level (SU, in mg/dL) (**C**) mean estimated glomerular filtration rate (eGFR, in mL/min/1.73 m^2^) of SPIRIT study participants at baseline, 3 months, 6 months, and 12 months, by treatment arm—SD (self-directed weight loss), CD (coach-directed weight loss), or met (metformin). Other abbreviations: CI: confidence interval; eGFR: estimated glomerular filtration rate.

At baseline, mean (SD) BMI was 34.7 (4.9), 35.3 (4.9), and 34.9 (6.3) in the self-directed, metformin, and coach-directed arms of the trial, respectively (Table 2). Change in BMI differed by randomized arm (Table 2, Figure 1A). In the self-directed arm, there was no change in BMI at each time point. However, in the coach-directed arm, BMI changed by −1.09 (CI: −1.46, −0.73, *p* < 0.001) at 3 months and −1.02 (CI: −1.60, −0.45, *p* < 0.001) at 12 months. In the metformin arm, BMI changed by −0.49 (CI: −0.80, −0.17, *p* = 0.002) at 3 months and −1.14 (CI: −1.76, −0.52, *p* < 0.001) at 12 months.

### 3.3. Change in Serum Urate from Baseline

At baseline, mean (SD) SU level was 5.8 (1.3) mg/dL, 5.8 (1.2) mg/dL, and 5.3 (1.4) mg/dL in the self-directed, metformin, and coach-directed arms, respectively (Table 2). The self-directed arm showed a significant reduction in SU at 12 months, by −0.25 mg/dL (CI: −0.48, −0.01, *p* = 0.04) from baseline, with a trend in the same direction at 3 and 6 months (Figure 1B). In the coach-directed arm, SU increased at 3 months by 0.26 mg/dL (CI: 0.04, 0.47, *p* = 0.02) from baseline, but regressed toward baseline values and was no longer significant at 6 months and 12 months (Figure 1B). In contrast, the metformin treatment arm did not show significant changes in SU compared to baseline (Figure 1B). Importantly, neither the coach-directed arm nor metformin treatment arm showed significant reductions in SU at any time point (Figure S1). At 12 months, specifically, there was no significant change in SU level compared to baseline in the coach-directed arm or the metformin treatment arm (Figure 1B).

### 3.4. Change in Glomerular Filtration Rate from Baseline

At baseline, mean (SD) eGFR was 84.2 mL/min/1.73 m^2^ (17.2), 86.8 mL/min/1.73 m^2^ (19.1), and 83.6 mL/min/1.73 m^2^ (20.1) in the self-directed, metformin, and coach-directed arms, respectively (Table 2). In the self-directed weight loss arm, there was a significant increase in eGFR at 3 months, by 4.24 mL/min/1.73 m^2^ (CI: 0.58, 7.89, *p* = 0.02), and at 12 months, by 4.37 (CI: 0.67, 8.07, *p* = 0.02). There were no significant changes in eGFR in the coach-directed arm, but eGFR did increase by 3.28 mL/min/1.73 m^2^ (CI: 0.02, 6.54, *p* = 0.048) at 6 months in the metformin arm.

### 3.5. Differences in Serum Urate between Intervention Arms

We examined SU levels between arms overall and at each visit. Compared to the self-directed arm, the coach-directed arm increased SU by 0.32 mg/dL (CI: 0.03, 0.61; *p* = 0.03) at 3 months (Table 3). Interestingly, this effect was stronger after adjustment for BMI at each time point (coach-directed vs. self-directed between-group difference at 3 months after BMI adjustment: 0.38 mg/dL; CI: 0.08, 0.68; *p* = 0.01). However, these effects were attenuated with adjustment for eGFR at each time point (coach-directed vs. self-directed between-group difference at 3 months after BMI and eGFR adjustment: 0.29 mg/dL; CI: −0.01, 0.59; *p* = 0.05). In addition, this coach-directed vs. self-directed between-group difference in SU, attenuated over time (coach-directed vs. self-directed between-group difference at 12 months with no adjustment: 0.26 mg/dL; CI: −0.07, 0.59; *p* = 0.12). Overall, across all time points, the coach-directed arm increased SU by 0.30 mg/dL (CI: 0.05, 0.55; *p* = 0.02) compared to the self-directed arm.

### 3.6. Relationships between Change in Serum Urate with Changes in BMI and eGFR

Finally, we examined the relationship between change in SU level and changes in BMI and eGFR from baseline across all visits, regardless of study arm. Pooled across all study visits, the relationship between change in SU and change in BMI was not significant, although a negative trend was observed (Figure S2A). Pooled across all study visits, change in SU was negatively associated with change in eGFR (β = −0.02; *p* < 0.001). When change in SU was examined by level of change in BMI, there remained no significant relationship at any level of BMI (Table 4). In contrast, change in SU level was examined by category of change in eGFR, a decrease in eGFR was associated with a significant increase in SU, while an increase in eGFR was associated with a significant decrease in SU (Table 4, Figure S2B).

## 4. Discussion

In this randomized trial of cancer survivors, coach-directed weight loss did not reduce SU. In fact, coach-directed weight loss was associated with increased SU in the short-term, at 3 months. Mediation analyses indicate that this increase in SU was stronger after accounting for the effects of weight loss on SU; however, this effect was attenuated after accounting for eGFR. This suggests that interventions that reduce BMI can have variable effects on SU, due to eGFR changes that may not reflect the intended effects of BMI reduction. Although our study did not track clinical events such as gout flares, it questions weight loss as a strategy for short-term SU reduction, and highlights the need for further research in patients with gout.

To date, evidence on the relationship of weight with SU comes primarily from observational studies. Obesity has been associated with hyperuricemia and gout in a previous cross-sectional study [3], with the population attributable risk of hyperuricemia due to being overweight/obese estimated at 44% [25]. In a recent cohort study, DASH-style diet, reduction of alcohol intake, and reduction of diuretic use were found to prevent the majority of incident gout cases among men with normal weight or overweight, but not among men with obesity, leading the authors to conclude that men with obesity may not benefit from other modifications unless weight loss is addressed [26]. Weight reduction has been associated with reductions in SU in observational studies [7,8,9,10,11,12,13,14]. Whether this weight loss was intentional or unintentional was not considered. In one prospective study of male runners, longer running distance and greater fitness were negatively associated with risk of gout, but this relationship was no longer significant after adjustment for change in BMI, suggesting exercise associations were mediated by BMI [12]. Weight loss has been traditionally thought to reduce SU level by increasing renal fractional excretion of SU [13].

Few trials have examined the relationship of weight loss interventions with change in SU. Although the Multiple Risk Factor Intervention Trial (MRFIT) was a randomized trial, analyses did not compare SU levels across treatment arms, because some components of the multiple risk factor intervention (e.g., thiazides) affected SU; rather, analyses demonstrated an association between change in SU and BMI from baseline [14]. In fact, our findings are similar to a recent analysis of two weight-loss diets, which showed no difference in SU between calorie-restricted and non-calorie restricted diets on SU in participants with diabetes [16]. Among the other studies of non-surgical weight loss, prior studies relied on participants’ self-initiation and self-report of physical activity (e.g., self-reported number of kilometers ran per day) rather than a directed weight loss intervention by coaching. Importantly, one prospective study of bariatric surgery patients, SU did not change following pre-surgical diet- and exercise-based weight loss (6.39 mg/dL at baseline to 6.39 mg/dL after exercise-based weight loss) [7]. Even more notable, in this same study of bariatric surgery patients, SU rose significantly from 6.39 mg/dL to 7.40 mg/dL in the 2-week postoperative period, before starting to fall again and reaching 5.04 mg/dL one year after surgery [7]. This supports the observation in our study that weight loss may result in a short-term increase in SU that becomes less prominent over time. A better understanding is needed regarding the time course of changes in SU with weight loss, along with underlying mechanisms.

There are several potential biologic pathways by which SU might increase acutely after accelerated weight loss, including increased tissue breakdown, starvation, dehydration, or the weight loss itself [27]. First, in the post-operative period mentioned above [7], high turnover of adenosine triphosphate (ATP) may result in increased release of SU. Second, fluctuating renal clearance may also play a role. Glomerular filtration rate (GFR) is a known determinant of SU concentration as the kidney is responsible for 60–70% of urate excretion [28,29]. Fractional excretion of SU is lower in obese patients than in normal controls [13]. In the period during and following weight loss, the accumulated SU may be due to decreased kidney excretion, leading to a transient rise in SU levels. Indeed, in our study, we found that the rise in SU at 3 months among those assigned to coach-directed weight loss was partially explained by eGFR but not by BMI. Third, it is possible that sodium reduction might explain our findings. While we did not collect information on sodium intake among participants of the three arms, lower sodium intake can also acutely lower GFR and cause a rise in SU by volume contraction [30,31], or by increased secondary active transport of SU in the proximal tubule [32]. Fourth, the observed SU effects could reflect normalization of glucose and insulin levels as both excess circulating glucose and insulin have been shown to increase excretion of uric acid in physiology studies [33,34,35,36].

Metformin has been hypothesized to reduce SU via synthetic pathways by lowering circulating free fatty acids [21]. In SPIRIT it also reduced weight loss, which we hypothesized might be another mechanism for SU reduction. However, metformin also increases insulin sensitivity and higher glucose and insulin levels are inversely associated with SU in adults with diabetes [37]. Thus, the aggregate effects of metformin on SU have been conflicting [38]. Our study found no effect from metformin on SU levels. This differs from two studies, which demonstrated reductions in SU after daily metformin intake [21,39]. However, these studies varied from SPIRIT in key ways. The Barskova study enrolled a population with diabetes, while the Kryztek-Korpacka study included children and adolescents [21,39]. SPIRIT was limited to adult cancer survivors without diabetes. In addition, Kryztek-Korpacka did not randomize subjects. Finally, both studies did not report whether the observed changes in SU were clinically significant.

Our study has several limitations. First, the coach-directed weight loss intervention included dietary calorie restriction, increased calorie expenditure, and potential changes in diet, all of which are difficult to measure. In this context, observed changes in SU might have resulted from changes in several of these factors, rather than, or in addition to change in BMI. Second, baseline SU levels were relatively low, 5.8 mg/dL (SD 1.3). Prior studies of lifestyle interventions have shown greater reductions in SU level in populations with higher baseline SU level [40,41]. Third, gout status and gout medications were not determined at baseline and gout flares were not monitored during the study, as it was not a focus of the original trial. Fourth, study participants were cancer survivors without gout. Additional studies, ideally in a general population that included persons with gout, are needed. Fifth, SPIRIT’s exclusion based on a hemoglobin A1c ≥ 7% may have included some adults with undiagnosed diabetes. Finally, SPIRIT’s small sample size reduces the trial’s overall power.

Our study also has multiple strengths. First, as a randomized trial, SPIRIT reduced the likelihood of confounding. Second, the study population was diverse in race and sex. Third, rates of adherence and follow-up were high. Fourth, SU was measured in serum specimens at a central laboratory without a freeze thaw cycle.

Our study has clinical implications. Obesity is common among adults with gout [3]. While weight loss is advantageous for many conditions frequently associated with gout (diabetes, elevated cholesterol, hypertension), our study highlights that in the short-term it may increase SU. Furthermore, our study raises the concern that SU lowering should not be assumed as a given consequence following weight loss. If confirmed, these findings serve as a further impetus to proceed with early initiation of SU lowering therapy when recommending weight loss in gout patients. In the 2020 ACR guidelines, the expert panel rated the certainty of evidence of weight loss as “very low” due to small sample sizes and high risk of bias [42]. Our study adds to the existing pool of evidence by providing the results of a randomized, controlled trial. While our study did not focus on a population with gout, our findings support current recommendations that weight loss not be the sole approach to SU reduction.

In conclusion, in this randomized trial of cancer survivors without gout, reductions in BMI either increased or did not change SU, potentially due to effects on eGFR. These results do not support a focus on BMI reduction for SU reduction; however, replication of this study in persons with gout is warranted.

## Figures and Tables

**Table 1:** Baseline characteristics of participants in the Survivorship Promotion In Reducing IGF-1 Trial (SPIRIT).

Characteristic	Self-Directed	Coach-Directed	Metformin	All
*N*	40	39	42	121
Age, years	58.8 (8.5)	60.9 (9.7)	59.5 (9.0)	59.7 (9.0)
Female, *n* (%)	32 (80%)	32 (82%)	32 (76%)	96 (79%)
African American, *n* (%)	13 (45%)	17 (44%)	20 (48%)	46 (45%)
Systolic blood pressure, mm Hg *	118.2 (13.2)	118.0 (26.7)	115.3 (14.6)	117.1 (18.9)
Diastolic blood pressure, mm Hg *	70.3 (9.3)	68.8 (15.3)	68.7 (8.5)	69.3 (11.3)
Body mass index, kg/m^2^ **	34.7 (4.9)	35.3 (5.0)	34.9 (6.3)	34.9 (5.4)
Total cholesterol, mg/dL	188.6 (40.9)	189.3 (37.8)	183.5 (41.0)	187.0 (39.7)
HDL cholesterol, mg/dL	48.4 (11.5)	58.6 (18.6)	55.0 (14.0)	54.0 (15.4)
eGFR (CKD-EPI), mL/min/1.73 m^2^	84.2 (17.2)	83.6 (20.1)	86.8 (19.1)	84.9 (18.8)
Any alcohol use, % ***	18 (46%)	16 (44%)	27 (66%)	61 (53%)
Cancer category, *n* (%) ****				
Breast	22 (55%)	19 (49%)	27 (64%)	68 (56%)
Prostate	4 (10%)	3 (8%)	4 (10%)	11 (9%)
Colon	4 (10%)	3 (8%)	3 (7%)	10 (8%)
Thyroid	4 (10%)	3 (8%)	2 (5%)	9 (7%)
Endometrial	2 (1%)	3 (8%)	2 (5%)	7 (6%)
Number of cancers, *n* (%) ****				
Only 1	37 (93%)	34 (87%)	37 (88%)	108 (89%)
More than 1	3 (8%)	5 (13%)	5 (12%)	13 (11%)
Serum urate, mg/dL	5.8 (1.3)	5.8 (1.1)	5.3 (1.4)	5.6 (1.3)
High serum urate, %	35%	31%	21%	29%

* N for systolic and diastolic blood pressure is 120 (one measurement missing from the metformin arm). ** Baseline body mass index was based on the average of two weight measurements performed prior to randomization. *** N for alcohol use is 116 (39 self-directed, 36 coach-directed, 41 metformin). **** Less common cancers not represented. Abbreviations: CKD-EPI: chronic kidney disease-epidemiology collaboration equation; eGFR: estimated glomerular filtration rate; HDL: high density lipoprotein

**Table 2:** Change in serum urate level (mg/dL) and body mass index (kg/m^2^) in SPIRIT compared to baseline at 3 months, 6 months, and 12 months, by randomized arm and among all participants.

		3-Months		6-Months		12-Months	
Assignment	Baseline Mean (SD)	Difference (95% CI)	*p*	Difference (95% CI)	*p*	Difference (95% CI)	*p*
**Serum urate, mg/dL**							
Self-Directed	5.8 (1.3)	−0.07 (−0.28, 0.14)	0.52	−0.12 (−0.33, 0.08)	0.23	−0.25 (−0.48, −0.01)	0.04
Coach-Directed	5.8 (1.1)	0.26 (0.04, 0.47)	0.02	0.17 (−0.08, 0.42)	0.18	0.00 (−0.26, 0.26)	0.99
Metformin	5.3 (1.4)	0.16 (−0.04, 0.36)	0.12	0.16 (−0.07, 0.39)	0.16	0.07 (−0.17, 0.30)	0.58
All participants	5.6 (1.3)	0.11 (−0.01, 0.24)	0.08	0.07 (−0.07, 0.20)	0.32	−0.06 (−0.21, 0.08)	0.39
**Body mass index, kg/m^2^**							
Self-Directed	34.7 (4.9)	−0.24 (−0.47, −0.00)	0.048	−0.27 (−0.60, 0.05)	0.10	−0.14 (−0.59, 0.30)	0.53
Coach-Directed	35.3 (4.9)	−1.09 (−1.46, −0.73)	<0.001	−1.31 (−1.84, −0.77)	<0.001	−1.02 (−1.60, −0.45)	<0.001
Metformin	34.9 (6.3)	−0.49 (−0.80, −0.17)	0.002	−0.82 (−1.26, −0.38)	<0.001	−1.14 (−1.76, −0.52)	<0.001
All participants	34.9 (5.4)	−0.59 (−0.78, −0.41)	<0.001	−0.79 (−1.06, −0.53)	<0.001	−0.75 (−1.08, −0.43)	<0.001
**eGFR (CKD-EPI), mL/min/1.73 m^2^**							
Self-Directed	84.2 (17.2)	4.24 (0.58, 7.89)	0.02	2.78 (−0.66, 6.22)	0.11	4.37 (0.67, 8.07)	0.02
Coach-Directed	83.6 (20.1)	0.23 (−2.73, 3.20)	0.88	1.38 (−1.97, 4.73)	0.42	0.94 (−2.56, 4.44)	0.60
Metformin	86.8 (19.1)	1.40 (−0.56, 3.36)	0.16	3.28 (0.02, 6.54)	0.048	−0.00 (−3.32, 3.32)	1.00
All participants	84.9 (18.8)	1.99 (0.27, 3.71)	0.02	2.50 (0.56, 4.43)	0.01	1.81 (−0.25, 3.87)	0.09

Note that these estimates were generated with generalized estimating equation models and as a result differ slightly from the Figure.

**Table 3:** Difference in serum urate level (SU, in mg/dL), body mass index (BMI, in kg/m^2^), and estimated glomerular filtration rate (eGFR in mL/min/1.73 m^2^) between treatment arms at 3 months, 6 months, and 12 months, with and without adjustment for BMI and eGFR.

	3-Months		6-Months		12-Months		Combined *	
	Difference (95% CI)	*p*	Difference (95% CI)	*p*	Difference (95% CI)	*p*	Difference (95% CI)	*p*
**Effects on serum urate**								
Coach-Directed vs. Self-Directed	0.32 (0.03, 0.61)	0.03	0.30 (−0.01, 0.62)	0.06	0.26 (−0.07, 0.59)	0.12	0.30 (0.05, 0.55)	0.02
Metformin vs. Self-Directed	0.17 (−0.12, 0.46)	0.24	0.24 (−0.06, 0.54)	0.12	0.27 (−0.05, 0.59)	0.10	0.21 (−0.04, 0.46)	0.10
Metformin vs. Coach-Directed	−0.15 (−0.45, 0.15)	0.34	−0.07 (−0.41, 0.28)	0.70	0.01 (−0.35, 0.36)	0.97	−0.09 (−0.37, 0.19)	0.53
**Effects on BMI**								
Coach-Directed vs. Self-Directed	−0.86 (−1.29, −0.44)	0.00	−1.04 (−1.66, −0.41)	0.00	−0.89 (−1.61, −0.16)	0.02	−0.93 (−1.47, −0.39)	0.00
Metformin vs. Self-Directed	−0.24 (−0.64, 0.15)	0.23	−0.55 (−1.09, 0.00)	0.05	−1.00 (−1.77, −0.24)	0.01	−0.58 (−1.09, −0.08)	0.02
Metformin vs. Coach-Directed	0.62 (0.14, 1.10)	0.01	0.49 (−0.20, 1.19)	0.17	−0.12 (−0.97, 0.73)	0.78	0.34 (−0.29, 0.98)	0.29
**Effects on eGFR**								
Coach-Directed vs. Self-Directed	−4.86 (−12.24, 2.53)	0.20	−2.26 (−9.43, 4.91)	0.54	−4.29 (−12.10, 3.53)	0.28	−4.40 (−11.44, 2.63)	0.22
Metformin vs. Self-Directed	−0.36 (−7.48, 6.76)	0.92	2.97 (−3.95, 9.90)	0.40	−1.90 (−8.79, 5.00)	0.59	0.61 (−6.05, 7.27)	0.86
Metformin vs. Coach-Directed	4.50 (−4.18, 13.18)	0.31	5.23 (−2.81, 13.27)	0.20	2.39 (−6.40, 11.17)	0.59	5.02 (−3.21, 13.24)	0.23
**Urate adjusted for BMI**								
Coach-Directed vs. Self-Directed	0.38 (0.08, 0.68)	0.01	0.38 (0.06, 0.69)	0.02	0.32 (−0.02, 0.66)	0.06	0.36 (0.10, 0.62)	0.01
Metformin vs. Self-Directed	0.19 (−0.11, 0.48)	0.21	0.27 (−0.04, 0.59)	0.09	0.34 (−0.01, 0.68)	0.05	0.25 (−0.01, 0.50)	0.06
Metformin vs. Coach-Directed	−0.19 (−0.49, 0.11)	0.21	−0.10 (−0.44, 0.24)	0.56	0.02 (−0.33, 0.36)	0.93	−0.12 (−0.39, 0.16)	0.41
**Urate adjusted for eGFR**								
Coach-Directed vs. Self-Directed	0.23 (−0.06, 0.52)	0.11	0.27 (−0.02, 0.57)	0.07	0.19 (−0.14, 0.51)	0.26	0.23 (−0.02, 0.49)	0.07
Metformin vs. Self-Directed	0.12 (−0.15, 0.40)	0.38	0.26 (−0.02, 0.54)	0.07	0.18 (−0.13, 0.48)	0.25	0.18 (−0.05, 0.41)	0.13
Metformin vs. Coach-Directed	−0.11 (−0.41, 0.19)	0.47	−0.01 (−0.33, 0.30)	0.93	−0.01 (−0.36, 0.34)	0.96	−0.06 (−0.33, 0.22)	0.68
**Urate adjusted for BMI and eGFR**								
Coach-Directed vs. Self-Directed	0.29 (−0.01, 0.59)	0.05	0.35 (0.04, 0.65)	0.03	0.24 (−0.09, 0.58)	0.15	0.30 (0.04, 0.56)	0.02
Metformin vs. Self-Directed	0.14 (−0.14, 0.42)	0.33	0.30 (0.01, 0.59)	0.05	0.25 (−0.07, 0.56)	0.13	0.22 (−0.02, 0.46)	0.08
Metformin vs. Coach-Directed	−0.16 (−0.46, 0.15)	0.32	−0.05 (−0.36, 0.27)	0.76	0.00 (−0.34, 0.34)	0.99	−0.08 (−0.36, 0.19)	0.55

* All analyses used xtgee adjusted for baseline urate. The combined analysis treated all visits equally, excluding baseline.

**Table 4:** (Top) Cross-sectional association of category of body mass index (BMI) change from baseline with serum urate (SU) change from baseline. *N* = 118 participants with 345 follow-up visits (Bottom) Cross-sectional association of category of estimated glomerular filtration rate (eGFR) change from baseline with SU change from baseline. *N* = 118 participants with 345 follow-up visits. The referent group was based on where the observed peak for BMI or a mid-point for eGFR. These comparisons were performed using generalized estimating equations.

Category of Change in BMI (kg/m^2^)	*N* of Follow Up Visits	β (95% CI) [Change in SU by Category]	*p*
Decrease ≥2	60	−0.09 (−0.41, 0.24)	0.60
Decrease ≥1 & <2	71	−0.12 (−0.41, 0.18)	0.45
Decrease ≥0.5 & <1	46	−0.13 (−0.41, 0.15)	0.37
No change (difference between 0.5 & −0.5)	103	−0.14 (−0.38, 0.11)	0.27
Increase ≥0.5 & <1	34	Reference	Reference
Increase ≥1	31	−0.13 (−0.44, 0.19)	0.43
**Category of Change in eGFR (mL/min per 1.73 m^2^)**	***N* of Follow-Up Visits**	**β (95% CI)** **[Change in SU by Category]**	***p***
Decrease ≥10	31	0.24 (0.05, 0.44)	0.014
Decrease ≥5 & <10	36	0.09 (−0.16, 0.33)	0.49
Decrease ≥0 & <5	86	Reference	Reference
Increase >0 & <5	88	−0.04 (−0.21, 0.13)	0.64
Increase ≥5 & <10	52	−0.34 (−0.55, −0.13)	0.002
Increase ≥10	52	−0.57 (−0.80, −0.33)	<0.001

## Data Availability

Data available upon reasonable request to corresponding author.

## Supplementary Materials

The following are available online at https://www.mdpi.com/article/10.3390/nu13082673/s1. Figure S1: Kernel density plots depicting (A) the probability density of serum urate level with time at baseline, 3 months, 6 months, and 12 months, (B) the probability density of all three follow-up visit serum urate levels by intervention arm, namely self-directed weight loss, coach-directed weight loss, and metformin, and (C) the probability density of change in serum urate level from baseline (Bl) by intervention arm, Figure S2: (A) Cross-sectional relationship between change in serum urate (SU) and body mass index (BMI) from baseline, pooled across all study visits, using a Lowess smoother. (B) Cross-sectional relationship between change in SU and estimated glomerular filtration rate (eGFR) from baseline, pooled across all study visits, using a Lowess smoother (Both), Table S1: Inclusion and exclusion criteria for the SPIRIT trial.
