# Supplementary material for: Effects of a Behavioral Weight Loss Intervention and Metformin Treatment on Serum Urate: Results from a Randomized Clinical Trial
Source: Nutrients. 2021 Jul 31;13(8):2673. doi: 10.3390/nu13082673 (PMC8401432; doi:10.3390/nu13082673)
Supplement: Supplementary file 1 [file nutrients-13-02673-s001.zip › SPIRIT Uric Acid 210520 Supporting Information.pdf]

Supporting Figures

Figure S1: Kernel density plots depicting (A) the probability density of serum urate level with time at baseline, 3 months, 6 months, and 12 months, (B) the probability density of all three follow-up visit serum urate levels by intervention arm, namely self-directed weight loss, coach-directed weight loss, and metformin, and (C) the probability density of change in serum urate level from baseline (Bl) by intervention arm.

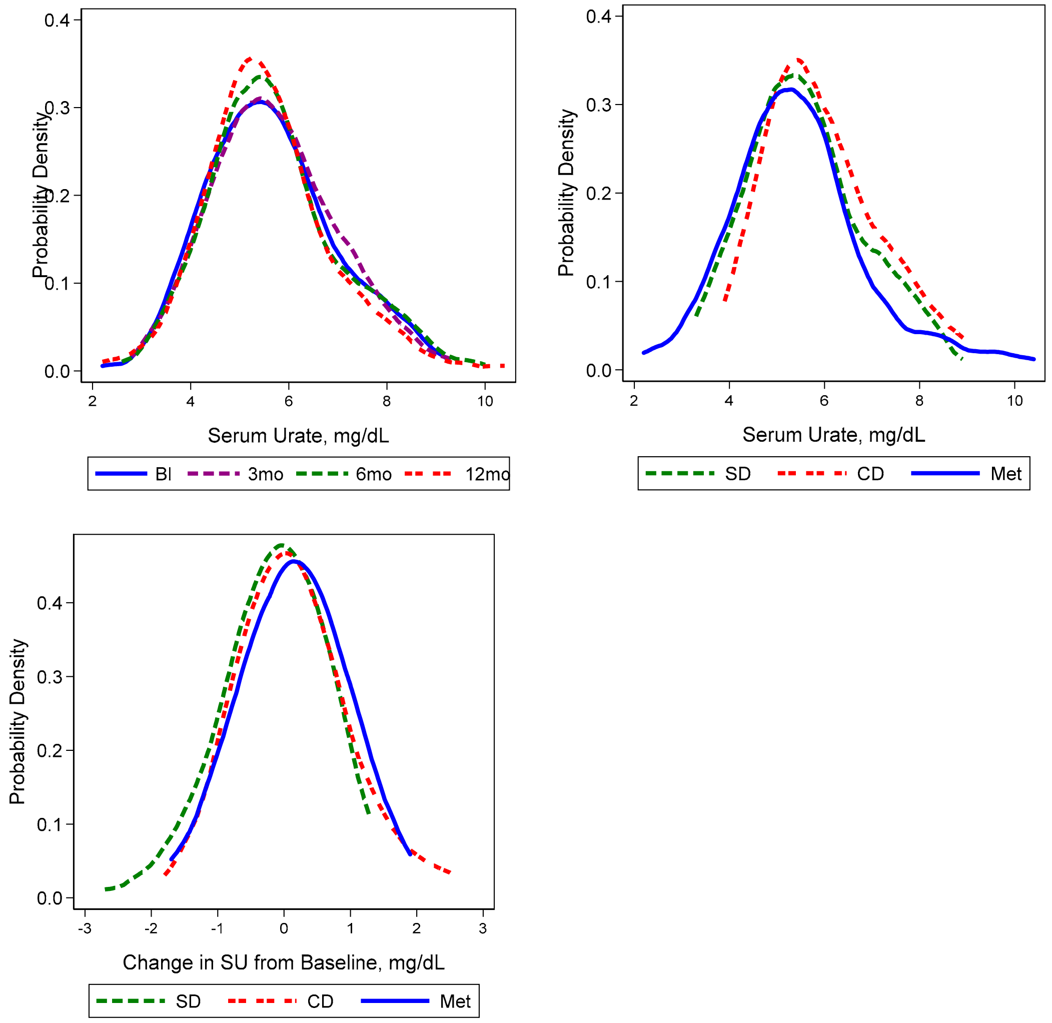

Figure S2: (A) Cross-sectional relationship between change in serum urate (SU) and body mass index (BMI) from baseline, pooled across all study visits, using a Lowess smoother. (B) Cross-sectional relationship between change in SU and estimated glomerular filtration rate (eGFR) from baseline, pooled across all study visits, using a Lowess smoother (Both). The x-axis depicts the either change in BMI from baseline, in kg/m^2, or change in eGFR from baseline, in ml/min/1.73 m^2. The y-axis depicts the change in SU from baseline, in mg/dL. Each open circle in the scatter plot denotes one patient follow-up visit. The red horizontal line represents no change. The blue line reflects the slope of a simple linear regression (Beta). The dashed black line is a model fit with a Lowess smoother.

(A)

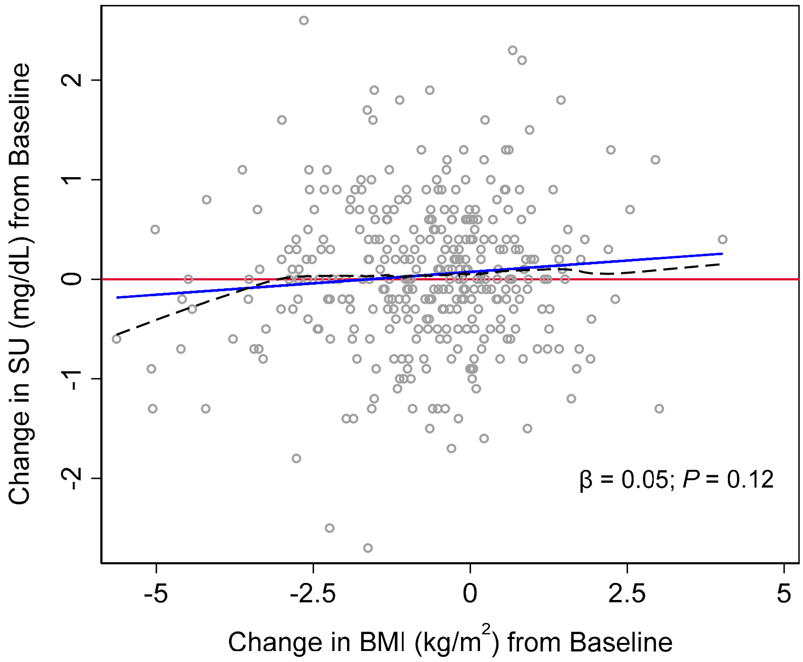

(B)

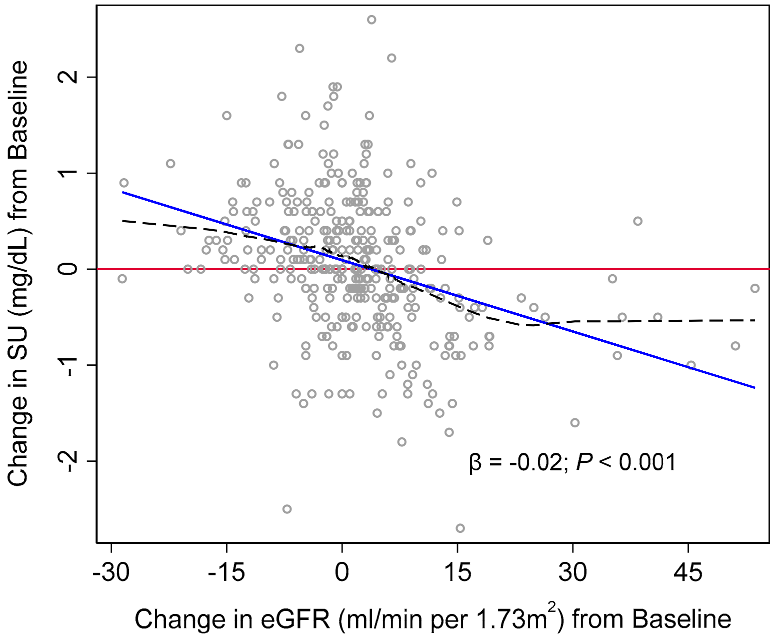

Change in SU (mg/dL) from Baseline
2
1
0
-1
-2
β = -0.02; P < 0.001
-30
-15
0
15
30
45
Change in eGFR (ml/min per 1.73m2) from Baseline

Supporting Tables

Table S1. Inclusion and exclusion criteria for the SPIRIT trial

Inclusion Criteria	Exclusion Criteria
• Women and men ages 18 or older<br>• Have been previously diagnosed with a malignant solid tumor, completed their required surgical, and/or chemotherapy and/or radiation curative intent therapy at least three months prior to enrollment, and have an anticipated treatment-free life span of 12 months or longer. Chemoprophylaxis with tamoxifen or aromatase inhibitors for breast cancer in women and anti-LHRH therapy for prostate cancer in men will be permitted.<br>• Have a BMI of 25 kg/m^2 or greater and weight <=400 lbs.<br>• Willingness to accept randomization to each of the three arms<br>• Willingness to change diet, physical activity, and weight<br>• Regular access to computer with a reliable Internet connection<br>• Ability to send and receive emails<br>• Ability to complete online forms<br>• Access to phone<br>• Willingness to provide written informed consent	• Women who are breastfeeding, pregnant, or planning pregnancy within the next year<br>• Medication-treated diabetes<br>• Fasting blood glucose >=200 mg/dL, or fasting blood glucose >=126 and <200 mg/dL and HbA1C >=7%<br>• Current or prior regular use of metformin within the past 3 months<br>• Uncontrolled concurrent medical condition likely to limit compliance with the study interventions<br>• Received any chemotherapy (unless anti-hormonal therapy) and/or radiation three months or less prior to the proposed intervention date<br>• Have a prior history of lactic acidosis by self-report<br>• Prior or planned bariatric surgery<br>• Have significant renal disease or dysfunction defined as eGFR<45<br>• Have significant hepatic dysfunction (AST/ALT ≥ 2 x ULN or reported liver disease)<br>• Self-reported average consumption of > 14 alcoholic drink per week<br>• Currently enrolled or planned to enroll in weight loss program<br>• Hemoglobin <9 g/dl<br>• Platelet count <100<br>• WBC <2.5<br>• Plans to relocate from the area within one years<br>• Use of prescription weight loss medication(s) (e.g., lorcaserin, topiramate/phentermine, phentermine, liraglutide, and bupropion/naltrexone), including off label use of drugs for

	weight loss or over-the-counter weigh loss medications such as Orlistat within the past 6 months.
